# Incidence and Related Factors of Infidelity among Medical Doctors and Nurses

**DOI:** 10.3390/ijerph18115575

**Published:** 2021-05-23

**Authors:** Sara Guerrero, Gracia Castro-Luna, Rosa Zapata Boluda, Aida Freites, Rafael García, Tesifón Parrón-Carreño

**Affiliations:** 1 Faculty of Health Sciences, Human Sexuality Institute, Autonomous University of Santo Domingo, Santo Domingo 10103, Dominican Republic; aidafreites@hotmail.com (A.F.); raf.garcia@hotmail.com (R.G.); 2 Faculty of Health Sciences, University of Almeria, 04120 Almeria, Spain; graciacl@ual.es (G.C.-L.); rzapata@ual.es (R.Z.B.); tpc468@ual.es (T.P.-C.)

**Keywords:** extra-dyadic sexual involvement, infidelity, shift work, doctors, nurses, health care personnel

## Abstract

Although there is a large body of research addressing infidelity, no study, to our knowledge, has specifically addressed infidelity in doctors and nurses and the correlation with work hours, schedule and other variables. This research aimed to know the incidence of and factors related to infidelity among doctors and nurses. A descriptive study was carried out, studying the association of certain variables. In total, 367 volunteer participants completed an online survey. Of them, 21% either have or have had an unfaithful relationship. The majority (81.7%) were doctors. Men were 4.3 times more unfaithful than women, with these differences being statistically significant (OR = 4.37, *p* < 0.001). Of the participants involved in an unfaithful relationship within the work area, the majority were men. Likewise, those who reported having had sex in the doctor’s room on duty were also men, with these differences being statistically significant (OR = 12.81, *p* < 0.01). The night emergency schedule was 60% more frequent in unfaithful people, and these differences were statistically significant (OR = 12.43, *p* < 0.01). There is a significant rate of infidelity in doctors and nurses. Men are more likely to be unfaithful than women are, and people who work nighttime emergencies are more likely to be unfaithful.

## 1. Introduction

Infidelity is one of the most complex and difficult to define areas in relationship studies. Riso defines it as the lack of the normative pact that limits the number of people involved in love or an erotic relationship and, therefore, the prohibition of maintaining others in parallel, whether occasional or continuous [1].

For Zumaya, the definition of infidelity is linked to the conception of a couple—if this concept were understood as the existence of a loving feeling and a commitment to emotional/erotic exclusivity between two individuals, infidelity would be a secret interpersonal relationship with a person outside of the couple with whom emotional and attraction elements of eventual or continuous occurrence are involved [2].

The value of fidelity has been of most importance for men and women. The violation of this pact of sexual and emotional exclusivity can cause different conflicts. In some situations, the solution to them can be conceived as separation. However, it is necessary to consider that the value attached to infidelity depends on the social perception, since an event can be interpreted as a transgression by certain people, whereas for others, it does not imply the breaking of the agreed rules. In this sense, social perception is conceptualized as the “awareness that an individual may have about the phenomena that occur around him, especially his ability to infer motivations, attitudes or values from the social behavior of others individuals [3]. Researchers have approached its origin from various areas, including type of attachment [4,5], anthropology [6,7], culture [8], personality [9] and gender [10]. Similarly, infidelity has been studied from a wide variety of perspectives, such as the reasons, types, educational level, gender differences, treatment, personality and occupation, among others. According to data provided by the Family Institute of the Dominican Republic, the number of cases of infidelity in couples has risen to 40% [11]. Likewise, according to the National Statistical Office of the Dominican Republic (ONE) data, 24,772 couples were divorced in the Dominican Republic in 2018. They found that frequent infidelity and financial situation were the main factors that led them to separate [12]. Kachadourian et al. studied whether distance from the partner for work reasons (in the military) could be related to infidelity. Of their study population, 22.2% reported that their partners were unfaithful to them during their absence [13]. Likewise, Snyder et al., in a study with the military, stated that the evidence suggests that in this population, especially in those who are separated from their partner for work missions, the rates of infidelity are significantly high [14]. Amocky et al. reported that high anxiety and stress levels could be a predictor of engaging in unfaithful behavior [15]. Romero highlights the influence of culture in the conceptualization of infidelity and the perceived consequences [16]. A higher frequency and acceptance of infidelity has been found in men than in women [17]. Atkins et al. found that people with a higher education level are more likely to engage in extramarital sex. They concluded a significant relationship between divorce and educational level, and the correlation between education and infidelity was only significant in divorced couples [18]. Labrecque and Whisman conducted a study based on nine years of research and interviews with 13,030 adults across the United States. This study reported that 53.3% of those surveyed who acknowledged having been unfaithful affirmed that they had sexual relations with someone they knew very well as a close friend, while 29.4% did it with close people, such as a neighbor or a co-worker. Furthermore, 21% of the men and 13% of the women interviewed admitted that they were unfaithful at least once in their life [19]. Takase stated that nursing has suffered from the general stereotype for a long time [20]. One of the causes attributed to the consolidation of the nursing image that has little to do with current reality is the hierarchical structure between the doctor and the nurse, which emanates from their respective educational origins [21]. Parera, Jorgelina et al. conducted a study on sexuality in health personnel. They stated that the practice of health-related professions is associated with physical and psychological exhaustion capable of producing alterations in the level of sexuality. Through a structured and anonymous survey, they evaluated the degree of satisfaction with the quality of sexual life and its factors in doctors, residents and nurses. Of the participants, 62% affirmed that since the beginning of the work activity, the frequency and quality of their sexual relations had decreased; 31% reported more significant attraction to other people and 15% reported infidelity [22]. The main objective of this work was to understand the high incidence of infidelity among health care workers. There is no study specifically on infidelity in health care workers, although there is such research in long-term jobs such as the military. The findings of this study will contribute to a better understanding of this phenomenon for both future research and therapeutical purposes.

## 2. Materials and Methods

### 2.1. Study Design

Cross-sectional descriptive study.

### 2.2. Study Population

The study population consisted of all the doctors and nurses who participated in the research, answering an anonymous online survey where questions were included to evaluate the study variables. The study sample consisted of 367 participants of different nationalities. As the majority of the responses were from the Dominican population (89.4%), with a minimal number of many other nationalities, that could contribute more bias of the study than benefits; it was preferred not to include it in the analysis. The survey was posted on an online portal so that health professionals from different parts of the world could have access to it and thus enrich the variety of responses, covering different nationalities and cultures. The Google survey system guarantees anonymity by not leaving any trace of who answered the survey and does not contain personal data that could allow the identification of any participant.

### 2.3. Ethical Criteria

All participants included voluntarily agreed to participate in this research; therefore, the international guidelines of the ethical protocols applied to research with human beings were taken into account.

### 2.4. Instrument

A survey was designed to include all of the information required to meet this research’s general and specific objectives; this survey was conducted in three languages Spanish, English and Portuguese. Native speakers of the three languages reviewed the versions in the corresponding language to ensure that the translation was adequate and precise for the participants [23].

### 2.5. Procedure

The invitation to participate in the study was sent via the Internet to doctors and nurses, who were asked to share it with colleagues, regardless of their location in the world. This way of collecting information was chosen because it effectively reaches more people in less time. In addition, it was carried out during the coronavirus pandemic, where there was social distancing.

### 2.6. Study Variables

The variables of the study were the following: age, sex, marital status, specialty, time spent with partner, number of previous marriages or stable relationships, time spent with the previous partner, work hours, with whom they have been involved, marital status of the person with whom they were unfaithful (if the person involved is related to the work area), who the person is with whom they were involved, type of unfaithful relationships, type of sexual exchange, place of meetings and sexual consequences, among others.

### 2.7. Analysis Plan

With the variables described above, a database was created, and their statistical treatment was carried out with SPSS version 24. A univariate analysis was performed. For the qualitative variables, frequencies with their corresponding percentages were calculated. For the quantitative variables, central tendency and dispersion measures (mean, minimum, maximum and standard deviation) were performed. In the bivariate analysis, for comparing qualitative variables, the chi-squared test (χ2) was applied, considering a value of *p* < 0.05 to be significant. The corresponding ORs were also calculated for 2 × 2 tables. For quantitative variables, a previous normality test (Kolmogorov–Smirnov) was used, as well as Student’s *t*-test (normal variables) and the Mann–Whitney U test (non-parametric variables). The multivariate analysis was performed using binary logistic regression, using infidelity as the dependent variable (yes/no), and as independent variables, all those that were significant in the bivariate analysis and those that were considered to influence the model. The Hosmer–Lemeshow goodness-of-fit test was performed, and a statistical significance level of *p* < 0.05 was considered to include variables in the final model.

## 3. Results

The mean age of the participants was 41.88 (12.52) years, with the youngest being 22 years old and the oldest being 78 years old.

The mean age of men, 45.94, was higher than that of women, 40.40, which was statistically significant (*p* < 001). Of those who were unfaithful, the mean age of men was 46.56 years and was also higher than that of women, 37.76 years, with this being a statistically significant difference (*p* < 001). The distribution by sex was 58 men (39.2%) and 90 women (60.8%). Of the participants, 21.5% had been in an unfaithful relationship.

People who worked nighttime emergency hours had the highest proportion of infidelity of 60%, and those who worked part-time had the lowest at 9.3%, with these differences being statistically significant (Table 1). No women had children, while seven men had created a child in the unfaithful relationship, and these differences were statistically significant (Table 2).

Statistically significant differences were found between the sex of the participant and whether or not they had had sexual encounters within the workplace. Of the participants who reported having had sexual encounters within the administrative offices, the majority (88.8%) were men; likewise, those who reported having had sexual encounters in the doctor’s room on duty (76.9%) were also mostly men. These differences were not statistically significant (Table 3).

Most of the women had relationships outside their work environment, although the differences were not statistically significant (Table 4).

For the multivariate analysis of binary logistic regression, infidelity was chosen as the dependent variable, and as independent variables, sex (1 man, 0 women), age, profession, specialty, marital status, time with partner, previous relationships and work hours were included.

A Nagelkerke R-squared value of 0.35 was obtained, and the Hosmer and Lemeshow test was performed with *p* = 0.35, indicating good fitted binary logistic regression model for the chosen analysis (Table 5).

The model only included the variable sex as significant, with an OR 4.73. Men were almost five times more unfaithful than women. Professionals who performed night emergencies showed 17.8 times more risk of being unfaithful than those who did part-time work.

## 4. Discussion

Infidelity in the professional environment is a highly publicized issue. Surprisingly, it has not been studied in the health care environment, where workers share very long working days—circumstances that, a priori, seem to favor this type of unfaithful behavior.

In this research, we have developed a binary logistic model that included the variable sex as significant, with an OR of 4.73. Men were almost five times more unfaithful than women, and it was observed that professionals who performed night emergencies had 17.8 times more risk of being unfaithful than those who did part-time work.

The results agree with those obtained by Buunk and Disjkstra, who stated that men are more likely to be unfaithful and involved in an unfaithful relationship than women [24].

Likewise, they also agree with the results of Caruso. Most of the investigations indicate that in urban Western societies, the ratio of infidelity in men and women is 3:1 or 2:1. These percentages increase when societies from third-world countries or rural communities are analyzed, reaching extreme situations in strongly religious countries where female infidelity has very low percentages or is almost non-existent [25].

Similarly, Camacho affirmed that infidelity is associated more with the male gender than with the female one. Men in all cultures and at all times were more unfaithful than women; that is a fact, although female infidelity is becoming more common [10]. Figueroa and Carolina studied sex differences in the motive of being unfaithful to a partner. Men were sex oriented and women were emotion oriented [26]. Similar results were reported by Jana Hackathon and Brian Ashdown about sexual vs. emotional motivation for infidelity [27]. Selterman et al., using an Internet-based questionnaire, found sexual desire, low commitment, lack of love and neglect as predictors of infidelity [28]. Fisher et al. published a review of the literature and studies on infidelity and reported that 1.5–4% of married men had had an extramarital relationship at some point in their life. They also reported that 23.2% of men are unfaithful to their current partners, and multiple studies reported that between 15% and 50% of men were unfaithful throughout their lives. These results are consistent with the results of our study, where it was found that men are more likely to be unfaithful than women (5.7 times), regardless of their marital status [29].

Concerning work hours, we found that night emergency hours were the most frequent in people who had been unfaithful. These differences were statistically significant.

Kachadourian et al. studied whether distance from the partner for work reasons (in the military) could be related to infidelity. Of their study population, 22.2% reported that their partners were unfaithful to them during their absence. Of the remaining participants, 37.8% admitted to being concerned about the possibility that their partner may have been unfaithful to them during their absence in combat. The participants who reported certainty of infidelity on the part of their partners exhibited high levels of symptoms due to post-traumatic stress and signs of severe depression compared to the individuals who did not report infidelity on the part of their partners. This correlates with our study questions, as doctors and nurses spend many hours away from their homes for work reasons [13].

We found that for the most part, the participants who had been unfaithful worked emergency hours at night. Likewise, Snyder et al., in a study with the military, stated that the evidence suggests that, in this population, especially in those who are separated from their partner for work missions, the rates of infidelity are significantly high [14].

Gordon and Mitchell studied whether the stress that the COVID-19 pandemic has brought has placed couples at higher risk of infidelity. The study was conducted across the United States and reported that people were engaging in behaviors highly related to being involved in an unfaithful relationship [30]. Fincham reported that infidelity is the most common cause of divorce [31].

Most of the participants who were involved with people not related to the institution were women. Some authors have reported the role of culture in the way infidelity is viewed depending on gender [16]. A higher frequency and acceptance of infidelity has been found in men than in women [17].

This could be due to the social stigma related to infidelity in women, since being involved with people outside of their work environment would guarantee greater discretion. Infidelity breaks gender expectations of what is culturally acceptable in terms of a woman’s sexual behavior and how others value her. This stigmatization could generate guilt, shame and fear.

These results are in line with what is expressed by Buss that men are not socially condemned for having this type of encounter. Even they allow it themselves without much guilt, whereas women, in general, are socially condemned for this or at least frowned upon, and they usually live with much guilt and many internal questions if they allow it [32].

Limitations: This study reveals different environmental layers of variables related to infidelity. Determining the effect size of the variables associated with infidelity was not possible due to the heterogeneity of the infidelity assessment tools. Infidelity will continue to be a challenge to the institution of marriage and couple relationships. New variables or factors that may influence infidelity behaviors such as social media, new technologies, a new perception of gender roles and family functions may require fresh attention from researchers to provide a new perspective on infidelity.

## 5. Conclusions

In conclusion, professionals in the field of medicine, who are subjected to many hours of work away from home, especially at night, have a significant incidence of infidelity. This incidence is higher in men. The motives given by participants to get involved in an extramarital relationship were related to the demands of their job, such as being away from home for many hours; being a way to release stress; and because of the nature of their work, it was easy to justify their absence from home. All of these data suggest that people working in the medical field could be more exposed to infidelity. These findings provide information that alerts this population to the importance of investing quality time in their relationship in their free time, given the nature of the work, the hours required and the access to private spaces suitable for sexual encounters. Having a better understanding of this phenomenon could reduce family conflicts related to this matter.

## Figures and Tables

**Table 1:** Demographic Characteristics.

			Infidelity		*p*-Value	OR IC 95%
			No	Yes		
**Sex**	Women	*n*	231	38	0.001	0.230.13–0.39
		%	85.9%	14.1%		
	Men	*n*	57	41		
		%	58.2%	41.8%		
Marital status	Romantic relationship	*n*	43	13	0.12	----
		%	76.8%	23.2%		
	Living together	*n*	34	17		
		%	66.7%	33.3%		
	Married	*n*	164	36		
		%	82.0%	18.0%		
	Single	*n*	47	13		
		%	78.3%	21.7%		
Work Schedule	Part-time	*n*	39	4	0.009	----
		%	90.7%	9.3%		
	Full-time	*n*	173	45		
		%	79.4%	20.6%		
	Unpredictable schedule	*n*	43	13		
		%	76.8%	23.2%		
	Full time and emergencies	*n*	29	11		
		%	72.5%	27.5%		
	Night emergencies	*n*	4	6		
		%	40.0%	60.0%		

**Table 2:** Distribution of the relationship of sex according to how many children have been created in unfaithful relationships.

	Men	Women	Total
None	31	38	69
One	7	0	7
More than one	3	0	3
Total	41	38	79

Likelihood ratio = 14.46; *p* = 0.001.

**Table 3:** Distribution of the relationship of sex according to whether the subject has had sexual encounters with people in the work area.

			Sex Inside the Work Area			Total
			Yes	Some of Them	No	
Sex	Women	*n*	8	14	16	38
		%	21.1%	36.8%	42.1%	100.0%
	Men	*n*	11	20	10	41
		%	26.8%	48.8%	24.4%	100.0%

Chi-squared 12.80; *p* = 0.24.

**Table 4:** Distribution of the relationship of sex according to where they have had sexual encounters within the work area.

	Men	Women	Total
Office and room of the doctors on duty	8	6	14
Room of the doctor on duty	13	7	20
Administrative office	8	1	9
None	9	13	22
Total	41	37	78

Likelihood ratio = 14.88; *p* = 0.061.

**Table 5:** Binary logistic regression analysis.

	OR	95% CI Para EXP (B)		*p*
		Lower	Higher	
Sex (men)	4.73	2.75	8.14	0.001
Work schedule (full-time work)	1.96	0.64	5.99	0.240
Work schedule (unpredictable schedule)	2.49	0.71	8.67	0.151
Work schedule (full-time and emergencies)	2.62	0.72	9.57	0.144
Work schedule (night emergencies)	17.79	3.34	94.87	0.001
Constant	0.07			

## Data Availability

Castro de Luna, Gracia (2021), “DATABASE INFIDELITY”, Mendeley Data, V1, doi:10.17632/j7vysf9kv8.1.
